# Targeted protein degradation in hematologic malignancies: latest updates from the 2023 ASH annual meeting

**DOI:** 10.1186/s13045-024-01533-w

**Published:** 2024-03-22

**Authors:** Guangcai Zhong, Ran Kong, Shi Feng, Cong Wang, Qingbo Hao, Weilin Xie, Xiangxiang Zhou

**Affiliations:** 1 Department of Hematology, Shandong Provincial Hospital Affiliated to Shandong First Medical University, No.324, Jingwu Road, Jinan, 250021 Shandong China; 2 Medical Science and Technology Innovation Center, Shandong First Medical University, Shandong Academy of Medical Sciences, Jinan, 250117 China; 3 Department of Hematology, Shandong Provincial Hospital, Shandong University, No.324, Jingwu Road, Jinan, 250021 Shandong China; 4 Institute of Materia Medica, Shandong First Medical University & Shandong Academy of Medical Sciences, Jinan, 250117 Shandong China; 5 National Clinical Research Center for Hematologic Diseases, The First Affiliated Hospital of Soochow University, Suzhou, 251006 China

**Keywords:** Protein degradation, Anti-tumor therapy, Hematologic malignancies

## Abstract

Protein degraders, emerging as a novel class of therapeutic agents, have gained widespread attention due to their advantages. They have several advantages over traditional small molecule inhibitors, including high target selectivity and ability to target “undruggable” targets and overcome inhibitor drug resistance. Tremendous research and development efforts and massive investment have resulted in rapid advancement of protein degrader drug discovery in recent years. Here, we overview the latest clinical and preclinical updates on protein degraders presented at the 2023 ASH Annual Meeting.

## To the editor

Small molecule inhibitors are major therapies used to treat hematologic malignancies in clinic, yet challenges such as drug resistance and off-target effects persist. Proteolysis-targeting chimeras (PROTACs) and molecular glues have emerged as promising therapeutic modality, celebrated for their ability to selectively target and efficiently degrade a wide array of proteins. This report summarizes the latest developments in protein degraders presented at 2023 ASH annual meeting.

## Clinical trials

Bruton’s tyrosine kinase (BTK), crucial for B-cell receptor signaling, has been identified as a key therapeutic target in B-cell malignancies. Three preliminary clinical trials assessed the safety and efficacy of BTK PROTAC degraders. In a phase 1 trial, BGB-16673, a potent BTK degrader, showed an overall response rate of 67% (12/18), including a mantle cell lymphoma patient achieving complete response (CR) after a median follow-up of 3.5 months in relapsed or refractory B-cell malignancies. The drug showed profound and enduring reductions of BTK protein even at the lowest doses. Moreover, although with 88.5% of patients experienced treatment-emergent adverse events (TEAEs), there were no discontinuations due to adverse events [1] (Table 1). NX-5948, a novel orally administered BTK PROTAC degrader, exhibited good tolerability with no dose-limiting toxicities or serious TEAEs. Following a median treatment duration of 2.8 months, 1 out of 3 evaluated chronic lymphocytic leukemia (CLL) patient achieved a partial response (PR) [2] (Table 1). NX-2127 is an oral dual-function PROTAC degrader targeting BTK and immunomodulatory factors Ikaros and Aiolos. NX-2127 showed potent and consistent degradation of target proteins. However, there were safety concerns, as some patients discontinued their treatment due to TEAEs. After a median follow-up of 9.5 months, lasting CR were noted in non-Hodgkin’s lymphoma patients [3] (Table 1).

**Table 1:** Clinical trials of protein degrader presented at ASH 2023

Compound	Modality	Target	Indication	Clinical Stage	Clinical Trial ID	Reference
BGB-16673	PRTOAC	BTK	R/R B-cell malignancies	Phase 1	NCT05006716	[1]
NX-5948	PRTOAC	BTK	R/R B-cell malignancies	Phase 1a/b	NCT05131022	[2]
NX-2127	PRTOAC	BTK/Ikaros /Aiolos	R/R B-cell malignancies	Phase 1a/b	NCT04830137	[3]
KT-333	PRTOAC	STAT3	R/R lymphoma, LGL-L, and ST	Phase 1a/b	NCT05225584	[4]

R/R, refractory/recurrence; LGL-L, large granular lymphocytic leukemia; ST, solid tumor; BTK: Bruton’s tyrosine kinase; STAT3: signal transducer and activator of transcription 3

Aberrant activation of phosphorylated signal transducer and activator of transcription 3 (STAT3) underlies various malignancies. KT-333, a STAT3 PROTAC degrader, achieved robust and dose-dependent degradation of STAT3 in a Phase 1a/1b study, with all the mean maximum degradation exceeding 60% across drug doses 1 to 4. Following two treatment cycles, one patient achieved PR. Importantly, no TEAEs were reported, suggesting a good safety profile of KT-333 [4] (Table 1).

## Preclinical studies

Several degraders targeting other pathways used alone or in combination, demonstrated good efficacy in pre-clinical tumor models. Many hematological malignancies feature the overexpression of anti-apoptotic proteins, such as BCL-2 and BCL-XL. Xie et al. reported that NXD02, a novel BCL-XL PROTAC degrader, displaying superior degradation of BCL-XL (DC50 of 6.6 vs. 17.4nM) and enhanced anti-tumor efficacy (100% vs. 36%) compared to DT2216, the initial BCL-XL PROTAC degrader [5](Table 2). The BCL-2/BCL-XL-dual degraders, WH25244 and PZ18753b, demonstrated impressive efficacy against venetoclax-resistant (VR) CLL cells [6] (Table 2). Additionally, combination therapy of DT2216 with azacitidine, ruxolitinib, or S63845, revealed synergistic effects against JAK2-mutant acute myeloid leukemia (AML) [7] (Table 2).

**Table 2:** Preclinical studies of protein degrader presented at ASH 2023

Compound	Modality	Target	Indication	Reference
NXD02	PROTAC	BCL-XL	AML	[5]
WH25244, PZ18753b	PROTAC	BCL − 2/ BCL-XL	VR CLL	[6]
DT2216	PROTAC	BCL-XL	JAK2-mut AML (post-MPN)	[7]
K-256	PROTAC	BET	MYC/BCL2-related lymphoma	[8]
KT-333	PROTAC	STAT3	VR AML, VR DLBCL	[9]
Mezigdomide	molecular glue	Aiolos/Ikaros	MM	[10]
PinA1	molecular glue	CK1α	AML	[11]

AML, acute myeloid leukemia; VR, venetoclax-resistant; CLL, chronic lymphoma leukemia; mut, mutation; MPN, myeloproliferative neoplasm; DLBCL, diffuse large B-cell lymphoma; MM, multiple myeloma; BET: Bromo and extra terminal;  STAT3: signal transducer and activator of transcription 3; CK1α: casein kinase 1α

In MYC/BCL2-related lymphoma, bromo and extra terminal (BET) inhibitors have shown limited efficacy in suppressing MYC expression. A novel BET PROTAC degrader K-256 effectively degraded bromodomain-containing protein 4 (BRD4) at low concentrations, leading to stronger tumor growth inhibition compared to existing inhibitors and other degraders [8] (Table 2).

STAT3 regulates anti-apoptotic protein MCL-1, which is implicated in VR AML. A STAT3 degrader significantly (> 60%) decreased MCL-1 and STAT3 levels, triggering apoptosis in VR AML cells. It also led to erythroid and myeloid differentiation induction of stem and progenitor cells [9] (Table 2).

Aiolos and Ikaros inhibit anti-tumor immunity through upregulating exhaustion markers in T cells, which blocking T cell activation and its tumor-killing activity. Mezigdomide, an Aiolos/Ikaros molecular glue degrader, effectively reversed the exhaustion of T cells, boosting the efficacy of immunotherapies in multiple myeloma [10] (Table 2).

Casein kinase 1α (CK1α), a multifunctional serine/threonine kinase, is involved in deactivating p53 function. PinA1, a selective CK1α molecular glue degrader, enhances p53 level and induces apoptosis in AML cells with wild-type TP53, while sparing normal cells. Additionally, PinA1 combined with FLT3, BCL-2, or MDM2 inhibitors showed enhanced anti-tumor effects in both in vitro and in vivo settings [11] (Table 2).

Molecular glue degraders such as pomalidomide and lenalidomide have demonstrated remarkable clinical success in treating hematological malignancies including multiple myeloma, and certain lymphomas. In addition to molecular glue, a lot of the recent efforts have been focused on developing PROTACs to overcome tumor resistance to inhibitor drugs. Early clinical studies and preclinical studies mentioned above have shown strong anti-tumor activities, both as standalone treatments and in combination therapies. These protein degraders showed their activities in overcoming resistance, reducing off-target effects, and enhancing treatment efficacy. Interestingly, concerns regarding the possibility of PROTACs resistance have promoted research into looking for alternative strategies, including the use of new E3 ligases other than only cereblon (CRBN) [12, 13]. While this is a very interesting concept, much more work is still needed to better define the strategy. We look forward to more comprehensive trial results that would provide further insights into the clinical utility of those novel protein degraders, and hope those degraders currently in preclinic stage will soon get into clinical trials where the therapeutic hypothesis of degrading those targets can be tested in clinic.

## Data Availability

No datasets were generated or analysed during the current study.

## Abbreviations

ASH: American Society of Hematology
PROTACs: Proteolysis-targeting chimeras
BTK: Bruton’s tyrosine kinase
CR: Complete response
TEAEs: Treatment-emergent adverse events
CLL: Chronic lymphocytic leukemia
STAT3: Signal transducer and activator of transcription 3
VR: Venetoclax-resistant
AML: Acute myeloid leukemia
BET: Bromo and extra terminal
BRD4: Bromodomain-containing protein
CK1α: Casein kinase 1α
CRBN: Cereblon
